# Dexmedetomidine may alleviate severe acute pancreatitis-associated lung injury by targeting the AIM2 inflammasome in endothelial cells

**DOI:** 10.1186/s12871-026-03917-6

**Published:** 2026-06-01

**Authors:** Jing Zhou, Peng Ge, Jinhua Cui, Guocheng Xuan, Chi Ma, Jiaqi Yao, Qingping Wen

**Affiliations:** 1 Department of Anesthesiology, First Affiliated Hospital of Dalian Medical University, Dalian, 116011 China; 2 Department of Anesthesiology, Xinhua Affiliated Hospital of Dalian University, Dalian, 116021 China; 3 Department of Interventional Therapy, First Affiliated Hospital of Dalian Medical University, Dalian, 116011 China; 4 Department of General Surgery, First Affiliated Hospital of Dalian Medical University, Dalian, 116011 China

**Keywords:** Acute pancreatitis, Lung injury, Dexmedetomidine, AIM2, Endothelial cells

## Abstract

**Background:**

Severe acute pancreatitis (SAP) is often associated with life-threatening acute lung injury (ALI), with its pathogenesis being intricately connected to dysregulated inflammatory responses. There is a deficiency of particular treatment options. Dexmedetomidine (DEX), a highly selective α2-adrenergic receptor (α2-AR) agonist, demonstrates not only sedative effects but also possesses anti-inflammatory and organ-protective properties. However, the mechanisms through which DEX exerts its effects in SAP and related pulmonary disorders remain uncertain.

**Methods:**

A rat SAP model was established via retrograde injection of 5% sodium taurocholate into the biliopancreatic ducts, with DEX intervention and positive control groups included. The effects of DEX on pathological damage to pancreatic and lung tissues, serum inflammatory factors, and pulmonary edema were evaluated in vivo. In vitro, lipopolysaccharide (LPS) stimulation of human umbilical vein endothelial cells (HUVECs) was used to model an inflammatory environment. Transcriptome sequencing, single-cell RNA sequencing data analysis, protein-protein interaction network construction, molecular docking, and molecular biology techniques were employed to investigate the action targets of DEX and its regulatory effects on the absent in melanoma 2 (AIM2) inflammasome signaling pathway.

**Results:**

DEX therapy attenuated the pathological injury to pancreatic and pulmonary tissues in SAP rats, decreased serum concentrations of amylase, interleukin-1 beta, and tumor necrosis factor-alpha, and suppressed pulmonary edema. Transcriptomic analysis revealed that DEX could partially reverse the disorder in lung tissue gene expression profiles induced by SAP and identified the “DEX target gene set”. Bioinformatics analysis identified AIM2 as the core target, and molecular docking indicated that DEX could bind to AIM2 effectively. Single-cell RNA sequencing analysis revealed that the target gene set was specifically highly expressed in endothelial cells. DEX inhibited the activation of AIM2 and Caspase-11, as well as the phosphorylation of the nuclear factor kappa-B signaling pathway in lung tissue and endothelial cells. It also decreased the expression of vascular cell adhesion protein-1 and matrix metalloproteinase-9 in lung tissue. In addition, AIM2 knocdown blocked LPS-induced apoptosis in HUVECs, and DEX showed no further inhibitory effect.

**Conclusion:**

This study identifies a novel mechanism in which DEX may alleviate pyroptosis of endothelial cells by targeting and inhibiting AIM2 inflammasome activation, thereby improving SAP-ALI.

**Supplementary Information:**

The online version contains supplementary material available at 10.1186/s12871-026-03917-6.

## Introduction

Acute pancreatitis (AP) represents a prevalent critical condition within the digestive system. The condition is characterized by inflammation resulting from the abnormal activation of pancreatic enzymes within pancreatic acinar cells, which leads to self-digestion, edema, hemorrhage, and necrosis [1]. While the majority of patients exhibit mild and self-limiting disease courses, around 20–30% will progress to severe acute pancreatitis (SAP). The lungs represent the most susceptible target organs among the distant organs implicated in SAP [2]. Acute pancreatitis-associated lung injury (APALI) is the most prevalent complication and the primary cause of mortality in the initial phase of SAP [3]. The pathological characteristics of APALI include disruption of the alveolar-capillary barrier, pulmonary edema, and a significant release of inflammatory mediators [4]. The comprehensive investigation of the pathogenesis of APALI, along with the identification of effective intervention targets, holds significant clinical relevance for enhancing the prognosis of patients.

In the clinical management of APALI, conventional fluid resuscitation, nutritional support, and the maintenance of organ function are critical components. Moreover, sedation and analgesia are essential elements, especially for critically ill patients who necessitate mechanical ventilation [5]. Dexmedetomidine (DEX) is commonly utilized in clinical environments due to its limited impact on respiratory function and its effective analgesic and anxiolytic characteristics [6]. Recent studies demonstrate that the clinical benefits of DEX encompass not only sedation and analgesia but also significant anti-inflammatory and organ-protective properties. Studies demonstrate that DEX mitigates inflammatory responses and tissue injury in sepsis, surgery, and ischemia-reperfusion through the inhibition of signaling pathways, such as NF-κB [7–10]. It is essential to clarify whether DEX can reduce AP and the related lung injury.

Dysregulated inflammation is the primary factor contributing to acute pancreatitis and its associated distant organ damage, with inflammasome activation serving as a crucial mechanism. The absent in melanoma 2 (AIM2) inflammasome has garnered considerable attention due to its capacity to identify double-stranded DNA within the cytoplasm [11, 12]. AP induces necrosis in pancreatic acinar cells, resulting in the release of damage-associated molecular patterns, including cellular DNA [13]. Recognition of these DNAs by AIM2 receptors in immune or parenchymal cells, including pulmonary vascular endothelial cells (ECs), leads to the formation of AIM2 inflammasomes, which subsequently activate caspase-1. Activated caspase-1 cleaves the precursors of interleukin-1β (IL-1β) and IL-18, leading to the generation of mature pro-inflammatory factors. Furthermore, it cleaves the Gasdermin D protein, resulting in a pro-inflammatory, lytic form of programmed cell death termed pyroptosis [14, 15]. Pulmonary vascular endothelial cells function as an essential barrier between blood and tissue; their pyroptosis leads to increased vascular permeability and enhanced infiltration of inflammatory cells, constituting a crucial mechanism in the development and progression of APALI [16]. The AIM2 inflammasome-mediated pyroptosis of endothelial cells is proposed as a crucial connection between localized pancreatic injury and the spread of pulmonary inflammation.

This research focused on developing a rat model of AP using the retrograde injection technique for the biliary and pancreatic ducts to evaluate the therapeutic efficacy of DEX at the animal level. Secondly, lipopolysaccharide (LPS) was employed to stimulate human umbilical vein endothelial cells (HUVECs), thereby simulating an inflammatory environment in vitro. Molecular biology techniques, including Western blot and immunohistochemistry, were utilized to assess the expression and localization of key proteins. Additionally, the signaling network of DEX and its influence on cellular heterogeneity were investigated through bioinformatics analyses, encompassing transcriptomics and single-cell RNA sequencing. This study investigated whether DEX alleviates SAP-induced endothelial damage by inhibiting pyroptosis and whether this effect is dependent on specific signaling pathways.

## Methods

### Materials and reagents

DEX was purchased from MedChemExpress in the United States. Dexamethasone, sodium taurocholate, and lipopolysaccharide were purchased from Sigma-Aldrich in the United States. HUVECs were purchased from Pricella Biotechnology. The rabbit anti-AIM2 polyclonal antibody, rabbit anti-caspase-11 polyclonal antibody, rabbit anti-phosphorylated P65 (p-P65), and rabbit anti-P65 were all purchased from Abclonal, China. The mouse anti-vascular cell adhesion molecule-1 (VCAM-1) monoclonal antibody and the mouse anti-matrix metalloproteinase-9 (MMP9) monoclonal antibody were purchased from Affinity in China.

### Animal grouping and treatment

Six- to eight-week-old male Sprague-Dawley rats (200–220 g) were obtained from the Laboratory Animal Center of Dalian Medical University. All animal experiment designs in this study have received approval from the University Laboratory Animal Research Ethics Committee of Dalian Medical University (AEE23098) and are done in compliance with AVMA Guidelines to minimize animal suffering to the greatest extent possible. Rats were acclimated under standard conditions (23–25 ℃, 12 h light/dark cycle) for one week before random assignment to four groups (*n* = 10): sham-operation (SO), SAP, DEX, and dexamethasone positive control (POS). The SAP model was established by retrograde injection of 5% sodium taurocholate (STC) solution into the biliary and pancreatic ducts. Briefly, rats were anesthetized with intraperitoneal injection of 2% pentobarbital sodium (3 mL/kg), placed in a supine position, and the abdominal skin was disinfected. The abdominal cavity was opened along the midline to expose the duodenum and associated ducts. The hepatic portal and duodenal ends of the biliary and pancreatic ducts were temporarily occluded with an arterial clip. A 1 mL syringe was used to puncture approximately 1 cm at the duodenal opening of the ducts, followed by injection of 5% STC (1.0 mL/kg) at a constant rate of 0.2 mL/min [17]. After injection, the occlusion was maintained for 3 min. The arterial clip was then removed, and successful modeling was confirmed by observing pancreatic congestion and edema. The abdominal incision was sutured in layers. The SO group underwent only laparotomy and abdominal closure. The DEX and POS groups received intraperitoneal DEX (10 µg/kg) and dexamethasone (2 mg/kg), respectively, immediately after modeling [18]. The SO and SAP groups received equivalent volumes of normal saline at corresponding time points.

All rats were euthanized via blood collection (5–10 ml) from the abdominal aorta under deep anesthesia (8% sevoflurane) 24 h post-modeling. Following the cessation of heart rate and respiration, the rats were observed for an additional 2 min to confirm death. Following sacrifice, pancreatic and pulmonary tissues were harvested. Serum was separated by centrifugation and stored at -80 ℃ for later analysis. Portions of tissue were fixed in 4% paraformaldehyde for paraffin embedding, sectioning, and histological examination. Selected lung tissues were used to determine the wet-to-dry weight ratio. Remaining tissues were immediately frozen in liquid nitrogen for subsequent molecular biological analyses.

### Cell culture and processing

HUVECs were cultured in DMEM high-glucose medium containing 10% fetal bovine serum in a 37 ℃ incubator with 5% CO₂. Cell passage is carried out when the density reaches 80%-90%, using 0.25% pancreatic enzyme-EDTA solution for digestion at 37 ℃ for 1–2 min, and passage is performed at a ratio of 1:2 to 1:4. To simulate the inflammatory environment in vitro and explore the protective mechanism of DEX, HUVECs were randomly divided into four groups: the control (CON) group, the LPS group, the DEX-M group and the POS group. The LPS group was cultured in a medium containing 1 µg/mL LPS for 6 h [19]. The LPS stimulation used here induces acute pyroptosis, whereas SAP-ALI in vivo involves a dynamic inflammatory process. This study focuses on endothelial responses during the peak inflammatory phase, which may not reflect mild or late-stage conditions. One h before LPS stimulation, the DEX and POS groups were pre-protected by adding medium containing 100 nM DEX or 100 nM dexamethasone. GeneChem (Shanghai) provided AIM2 siRNA (si-AIM2) and control siRNA. According to the instructions, HUVECs were incubated with the siRNA. The knockdown efficiency of AIM2 was detected by quantitative real-time polymerase chain reaction (qRT-PCR). The primer sequences of AIM2 were based on previous study and the knockdown efficiency of AIM2 was detected under LPS stimulation [20]. Both sequences 1 and 2 could significantly reduce the mRNA expression level of AIM2, and sequence 2 had a higher knockdown efficiency than sequence 1, although the difference was not statistically significant (Supplementary Figure S1). We then chose sequence 2 for functional validation. Collect cell precipitates for subsequent analysis.

### Histological observation

The fixed pancreatic and lung tissues were routinely embedded in paraffin, cut into 4 μm sections, and stained with hematoxylin and eosin (H&E). The degree of pancreatic tissue injury was evaluated based on a scoring system that included acinar cell vacuolization, edema, inflammatory infiltration, and necrosis (Each indicator scores 0–4) [21]. Lung tissue injury was scored based on the thickness of alveolar walls, alveolar cavity congestion and hemorrhage, inflammatory cell infiltration, and the degree of pulmonary interstitial edema (Each indicator scores 0–4) [17]. The assessment was independently completed by two pathology researchers who were unaware of the groupings.

### Detection of serum indicators

Serum amylase activity was measured using an automatic biochemical analyzer according to the manufacturer’s instructions. Serum concentrations of IL-1β and tumor necrosis factor-α (TNF-α) were determined by ELISA. Activities of alanine aminotransferase (ALT) and aspartate aminotransferase (AST) were also assessed.

### Wet-to-dry ratio determination

Immediately after the rats were sacrificed, a portion of the right lower lung tissue was excised. Surface moisture was removed with filter paper, and the tissue was weighed to obtain the wet weight. The tissue was then dried in a 70 ℃ oven for 48 h until a constant weight was reached, and the dry weight was recorded. The wet-to-dry (W/D) weight ratio of lung tissue was calculated.

### Immunohistochemistry

Sections of lung tissue were taken, and after dewaxing and hydration, antigen remediation was performed. Subsequently, endogenous peroxidase activity was blocked with 3% hydrogen peroxide, and the non-specific binding site was blocked with 5% bovine serum albumin. The sections were incubated overnight at 4 ℃ with anti-VCAM-1 and mouse anti-MMP9 primary antibodies, respectively. The next day, the corresponding secondary antibody labeled with HRP was incubated at room temperature for one h. DAB was used for color development, and the cell nuclei were re-stained with hematoxylin. Finally, the plates were sealed with neutral gum. Images were observed and collected under an optical microscope, and semi-quantitative analysis of the positive expression of VCAM-1 and MMP9 was performed using Image J software.

### Western blot

Total protein was extracted from frozen rat lung tissue or HUVECs using RIPA lysate, and the protein concentration was determined. Take equal amounts of protein samples for SDS-polyacrylamide gel electrophoresis separation, and then transfer them onto PVDF membranes. After being sealed at room temperature with TBST solution containing 5% skimmed milk for one h, the membrane was incubated overnight with the corresponding primary antibodies: AIM2, Caspase-11, p-P65, P65, Bax, Cleaved Caspase-3, Bcl-2, and the internal reference β-actin/GAPDH at 4 ℃. The next day, the PVDF membranes were incubated at room temperature with an HRP-labeled secondary antibody (1:5000) for one h, then developed with ECL chemiluminescence reagent. The gray value of the target band was analyzed by Image J software, and the relative expression level was represented by the ratio of the target protein to β-actin.

### Transcriptomic analysis

Total RNA was extracted from lung tissues of rats in the SO, SAP, and DEX groups (*n* = 3 per group), and transcriptomic sequencing (RNA-seq) was performed. The sequencing data were processed using the DESeq2 software package. Based on the criteria of log_2_(Fold Change) > 1 and adjusted P value < 0.05, differentially expressed genes (DEGs) between the SAP group vs. SO group and the DEX group vs. SAP group were screened. By conducting an intersection analysis of the DEGs from the DEX and SAP groups, the “DEX target gene set” was determined for subsequent analysis.

### Single-cell sequencing data analysis

To analyze the expression distribution of potential DEX targets at the cell subpopulation level, we downloaded the publicly available single-cell sequencing dataset (GSE276682, public on Oct 29, 2024) of ALI model mice from the GEO database [22]. Data preprocessing (including quality control and cell filtering, data standardization and integration, hypervariable gene screening and linear dimensionality reduction, cell clustering and nonlinear dimensionality reduction, and cell subpopulation annotation) was accomplished in the R language environment using the Seurat software package (v 4.4.0). Subsequently, calculate the score of the “DEX target gene set” in each cell, and visualize the distribution of this score in different cell subpopulations through a violin plot and feature plot to infer the main target cell types of DEX action.

### Enrichment analysis and PPI network construction

The “clusterProfiler” package in the R was used to conduct gene ontology (GO) functional annotation and Kyoto Encyclopedia of Genes and Genomes (KEGG) pathway enrichment analysis of the screened DEGs. Expression profile data for all genes were analyzed using Gene set enrichment analysis (GSEA) software. Meanwhile, the DEGs were uploaded to the STRING database to construct a protein-protein interaction (PPI) network, and the CytoHubba plugin in Cytoscape was used. Three algorithms, namely Edge Percolated Component (EPC), Maximum Group Centrality (MCC), and Maximum Neighborhood Component (MNC), are comprehensively adopted to screen the core nodes (hub genes) in the PPI network.

### Molecular docking

Obtain the crystal structure of the AIM2 protein from the PDB database. The 3D chemical structure of DEX can be downloaded from the PubChem database. Docking simulations were conducted using Discovery Studio 2021. Before docking, the protein receptor undergoes pretreatment such as dewatering and hydrogenation. Rigid docking was performed using the Libdock module, with DEX as the ligand, and the ligand was docked to the active pocket of the AIM2 protein. Evaluate the docking results based on the docking score and binding mode (especially hydrogen bonds and hydrophobic interactions), and visually analyze the binding sites.

### Statistical analysis

All experimental data are presented as mean ± standard deviation (x ± s). Statistical analyses were performed using R software (v4.4.0). One-way analysis of variance (ANOVA) was used for comparisons among multiple groups, and the LSD-t test was applied for pairwise group comparisons. Differences were considered statistically significant at P-value < 0.05.

## Results

### DEX alleviates pancreatic and lung tissue damage in SAP rats

**Fig. 1:**
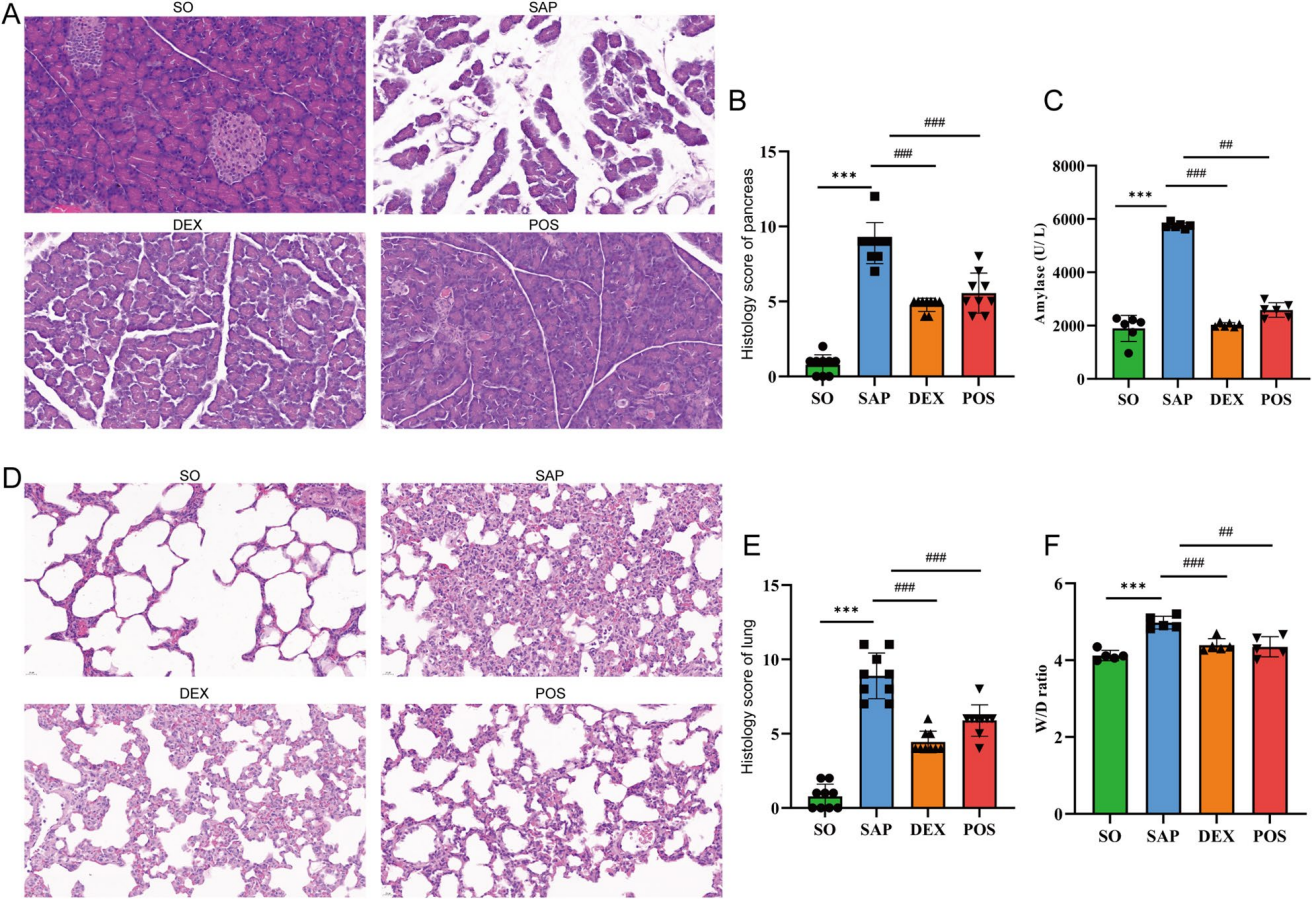
DEX reduces pancreatic and lung tissue damage in SAP rats. **A** Representative H&E-stained images of pancreatic tissue from each group (scale: 50 μm). **B** Quantification of pancreatic histopathological scores (*n* = 3). **C** Serum amylase levels in each group (*n* = 6). **D** Representative H&E-stained images of lung tissue from each group (scale: 50 μm). **E** Quantification of lung histopathological scores (*n* = 3). **F** Wet/dry weight ratio of lung tissue (*n* = 6). Data are presented as mean ± standard deviation. ***P-value < 0.001 vs SO group, ##*P*-value < 0.01 vs SAP group, ###*P*-value < 0.001 vs SAP group

The therapeutic effect of DEX on SAP was evaluated by assessing its impact on pathological damage in pancreatic and lung tissues. Pancreatic tissue in the SO group exhibited normal structure, whereas the SAP group demonstrated acinar cell necrosis, extensive inflammatory cell infiltration, and severe edema (Fig. 1A). DEX intervention ameliorated these pathological alterations. Statistical analysis of pathological scores (Fig. 1B) indicated that the pancreatic injury score in the DEX group was lower than in the SAP group. Serum amylase activity, which increased substantially in SAP rats, was significantly reduced following DEX treatment (Fig. 1C). In the SAP group, lung tissues displayed pronounced alveolar structural destruction, thickened alveolar walls, widespread inflammatory cell infiltration, and pulmonary edema (Fig. 1D). DEX administration mitigated these pathological changes, resulting in a lower pathological score compared to the SAP group (Fig. 1E). Furthermore, the wet/dry weight ratio of lung tissue, an objective measure of pulmonary edema, was elevated in the SAP group but was reduced in the DEX group (Fig. 1F).

### DEX inhibits the inflammatory response and liver injury in SAP rats

**Fig. 2:**
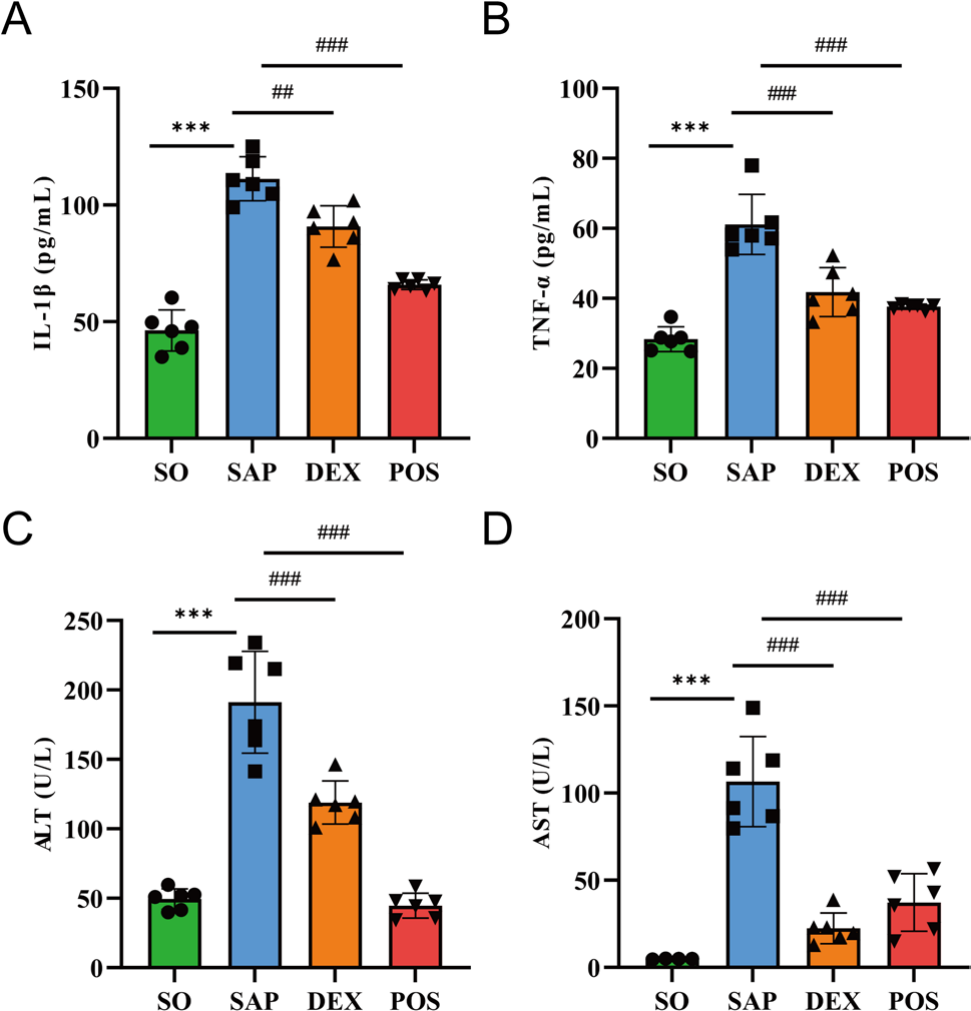
DEX inhibits the inflammatory response and liver injury in SAP rats. Serum levels of IL-1β (**A**), TNF-α (**B**), ALT (**C**), and AST (**D**) in each group (*n* = 6). Data are presented as mean ± standard deviation. ***P-value < 0.001 vs SO group, ##*P*-value < 0.01 vs SAP group, ###*P*-value < 0.001 vs SAP group

We further examined the inflammatory indicators. Compared with the SO group, serum levels of the pro-inflammatory cytokines IL-1β and TNF-α were increased in SAP rats (Fig. 2A&B). DEX treatment reduced the concentrations of these inflammatory mediators. Additionally, the systemic inflammatory response induced by SAP resulted in liver function impairment, as evidenced by an increase in serum ALT and AST activity. DEX intervention reversed these elevations (Fig. 2C&D).

### Identification of the targets for DEX intervention in SAP using omics data

**Fig. 3:**
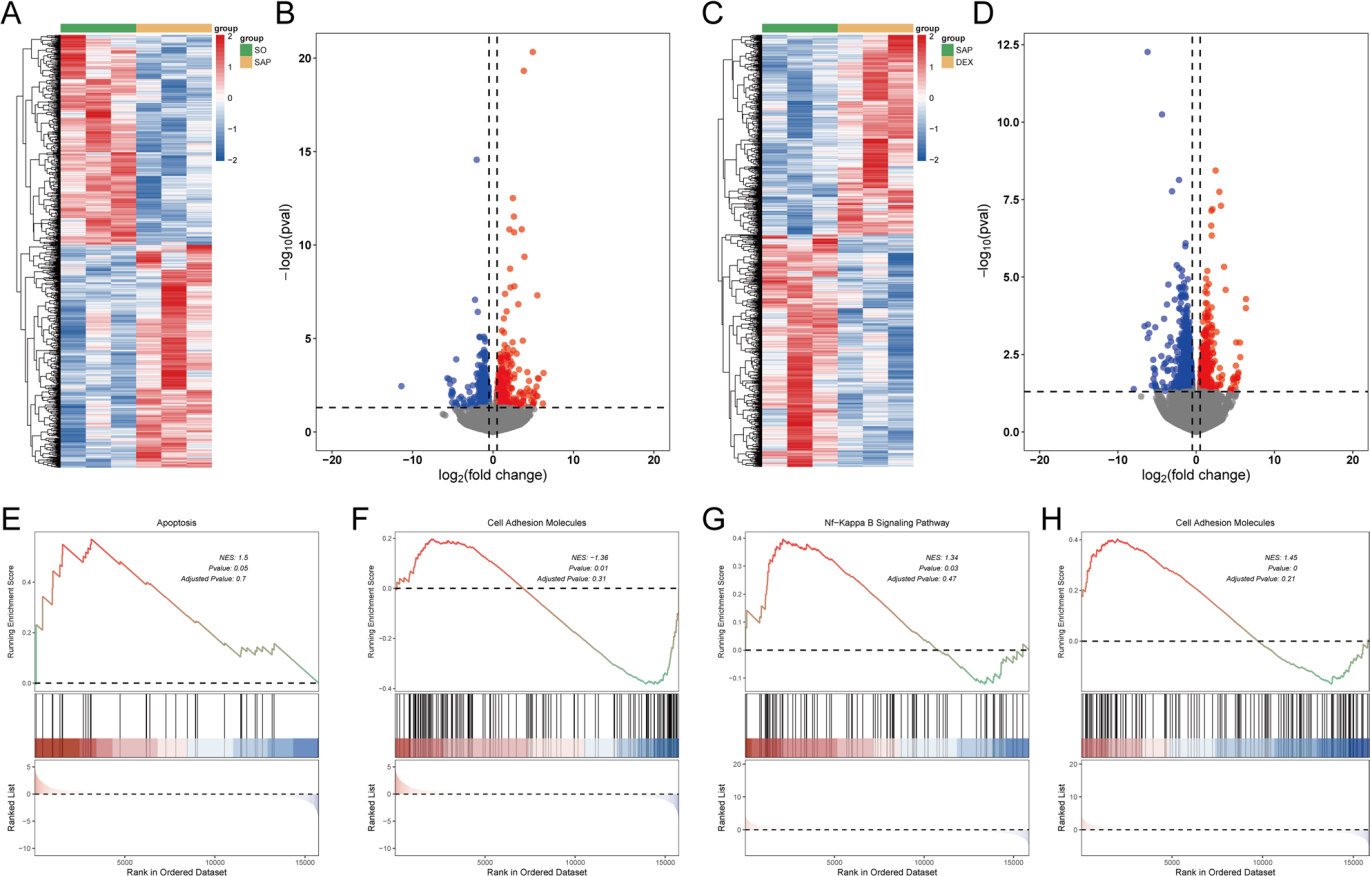
RNA-seq analysis of DEX effects on the lung tissue transcriptome in SAP rats. **A** Heat maps of gene expression in SAP and SO groups. **B** Volcano plots of differentially expressed genes between the SAP and SO groups. **C** Heat maps of gene expression in DEX and SAP groups. **D** Volcano plots of differentially expressed genes between DEX and SAP groups. **E**-**H** Representative GSEA enrichment pathway maps

To deeply explore the molecular mechanism of DEX, we performed transcriptomic sequencing of lung tissue. The heat map in Fig. 3A shows differences in gene expression profiles between the SAP and SO groups. The volcano plot in Fig. 3B shows that compared with the SO group, the SAP group has some differentially expressed genes (DEGs). It is worth noting that after DEX intervention, the gene expression profile underwent reprogramming, and the heat map was different from that of the SAP group (Fig. 3C). The volcano map (Fig. 3D) also showed that many genes were differentially expressed between the DEX and SAP groups. GSEA further indicated that DEX was enriched in multiple signaling pathways related to inflammation suppression and cell protection (Fig. 3E-H).

The intersection analysis of DEGs that experienced pullbacks after DEX intervention was conducted (Supplementary Figure S2A). The gene set was imported into the STRING database to construct a PPI network, and three algorithms (EPC, MCC, MNC) were used to identify core nodes. As shown in Supplementary Figure S2B-D, the three algorithms consistently identified 15 DEGs as the “DEX target gene set”. GO and KEGG enrichment analyses were conducted on the “DEX target gene set”. GO analysis (Supplementary Figure S3A) revealed that these genes were significantly enriched in biological processes, including inflammatory responses and pyroptosis. Among the first 15 pathways analyzed by KEGG Pathway (Supplementary Figure S3B), the NOD-like receptor signaling pathway, the Toll-like receptor signaling pathway, and the Cytosolic DNA-sensing pathway were significantly enriched. To further verify the interaction between DEX and the core target AIM2, we conducted molecular docking. The results showed that the DEX molecule could stably embed into the active pocket of the AIM2 protein and form good hydrogen bonds and hydrophobic interactions with the key amino acid residues (Supplementary Figure S3C).

### Single-cell RNA sequencing data analysis

**Fig. 4:**
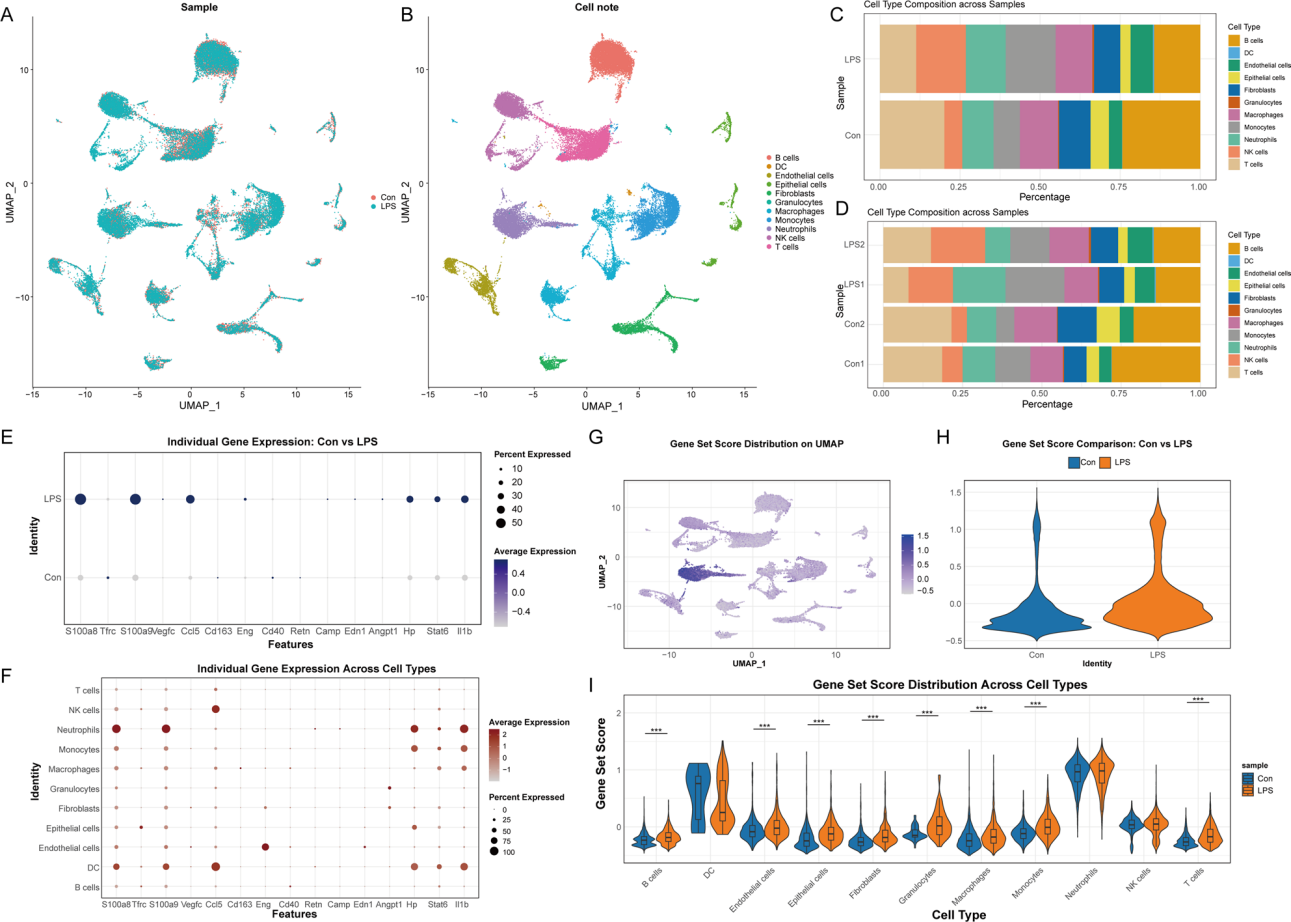
Single-cell RNA sequencing identifies cell subpopulations influenced by the DEX target gene set. **A** UMAP plots of all cells in the CON and LPS groups. **B** Annotated UMAP of cell subpopulations. **C** Stacked bar chart of cell subpopulation proportions in each group. **D** Changes in cell subpopulation proportions across samples. **E** Average expression of the DEX target gene set in different samples. **F** Average expression of the DEX target gene set in various cell subpopulations. **G** UMAP plot of DEX target gene set scores. **H** Comparison of gene set scores between CON and LPS groups. **I** Comparison of gene set scores across cell subpopulations

To identify potential DEX targets at the cell subpopulation level, public single-cell datasets were analyzed. Fig. 4A&B present the UMAP clustering and annotation results of lung tissue cells from mice with CON and acute lung injury induced by LPS. Cell proportion analysis showed a significant increase in immune cells, including macrophages, following LPS stimulation (Fig. 4C&D). Mapping the “DEX target gene set” to this dataset revealed upregulation of these genes after LPS stimulation (Fig. 4E), with predominant expression in ECs (Fig. 4F). Gene set score analysis further demonstrated that scores were significantly higher in endothelial cell subset than in other cell types (Fig. 4G&I) and increased in the LPS group, suggesting that these cells are primary responders to inflammation and potential core targets of DEX (Fig. 4H).

### DEX down-regulated the expressions of VCAM-1 and MMP9 in the lung tissues of SAP rats

**Fig. 5:**
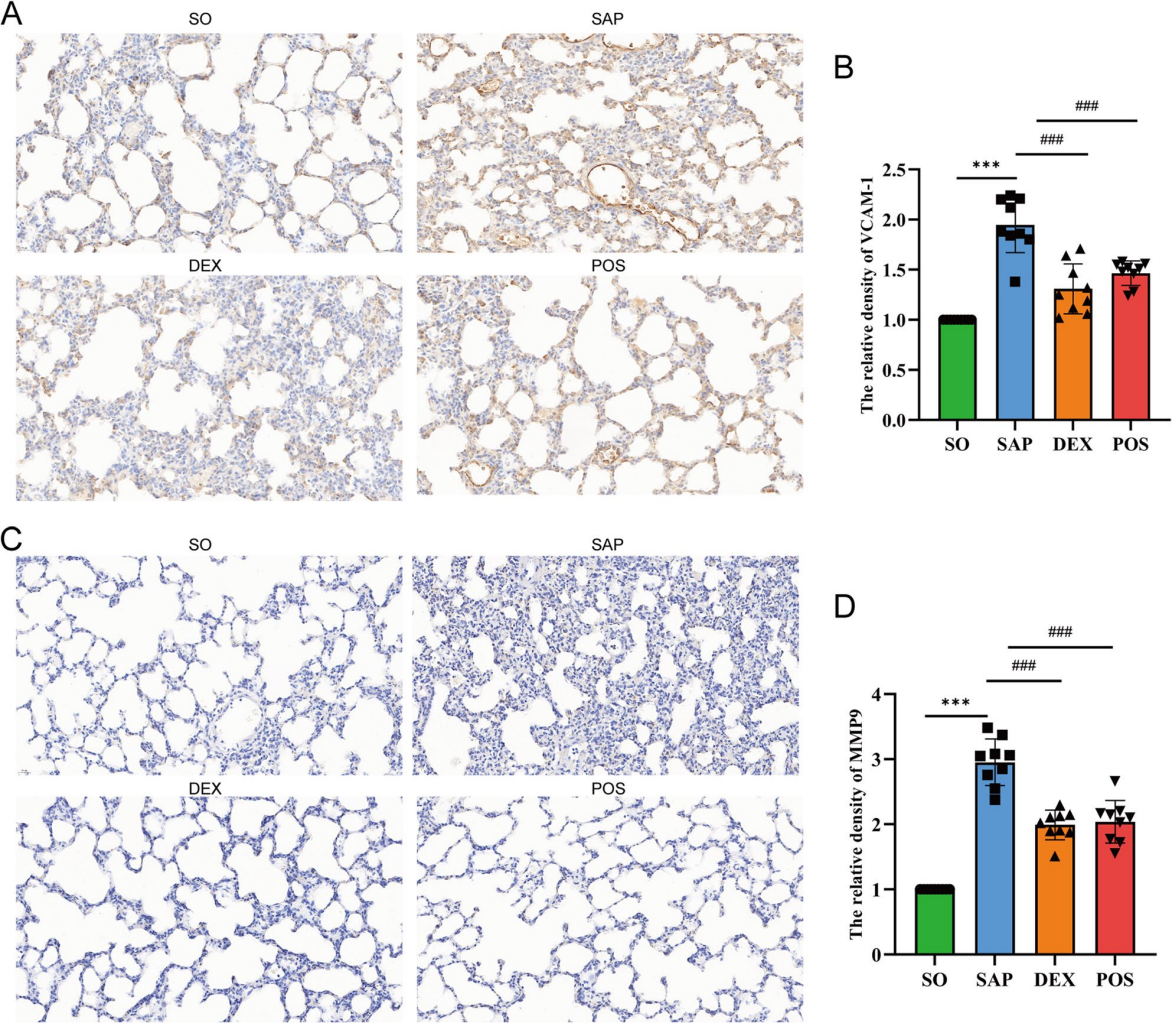
DEX regulates VCAM-1 and MMP9 expression in lung tissue from SAP rats. **A**, **B** Representative immunohistochemical images of VCAM-1 in lung tissue (scale: 50 μm) and corresponding semi-quantitative analysis (*n* = 3). **C**, **D** Representative immunohistochemical images of MMP9 in lung tissue (scale: 50 μm) and corresponding semi-quantitative analysis (*n* = 3). Data are presented as mean ± standard deviation. ^***^P-value < 0.001 vs. SO group, ^###^*P*-value < 0.001 vs. SAP group

The immunohistochemical results showed that, compared with the SO group, the expression of VCAM-1 and MMP9 in lung tissues of rats in the SAP group was increased. VCAM-1 is mainly expressed in ECs (Fig. 5A&B), while MMP9 is highly expressed in inflammatory cells and some structural cells (Fig. 5C&D). DEX treatment can inhibit the expression of these two proteins closely related to inflammatory infiltration and tissue remodeling.

### DEX inhibits the AIM2 inflammasome signaling pathway both in vivo and in vitro

**Fig. 6:**
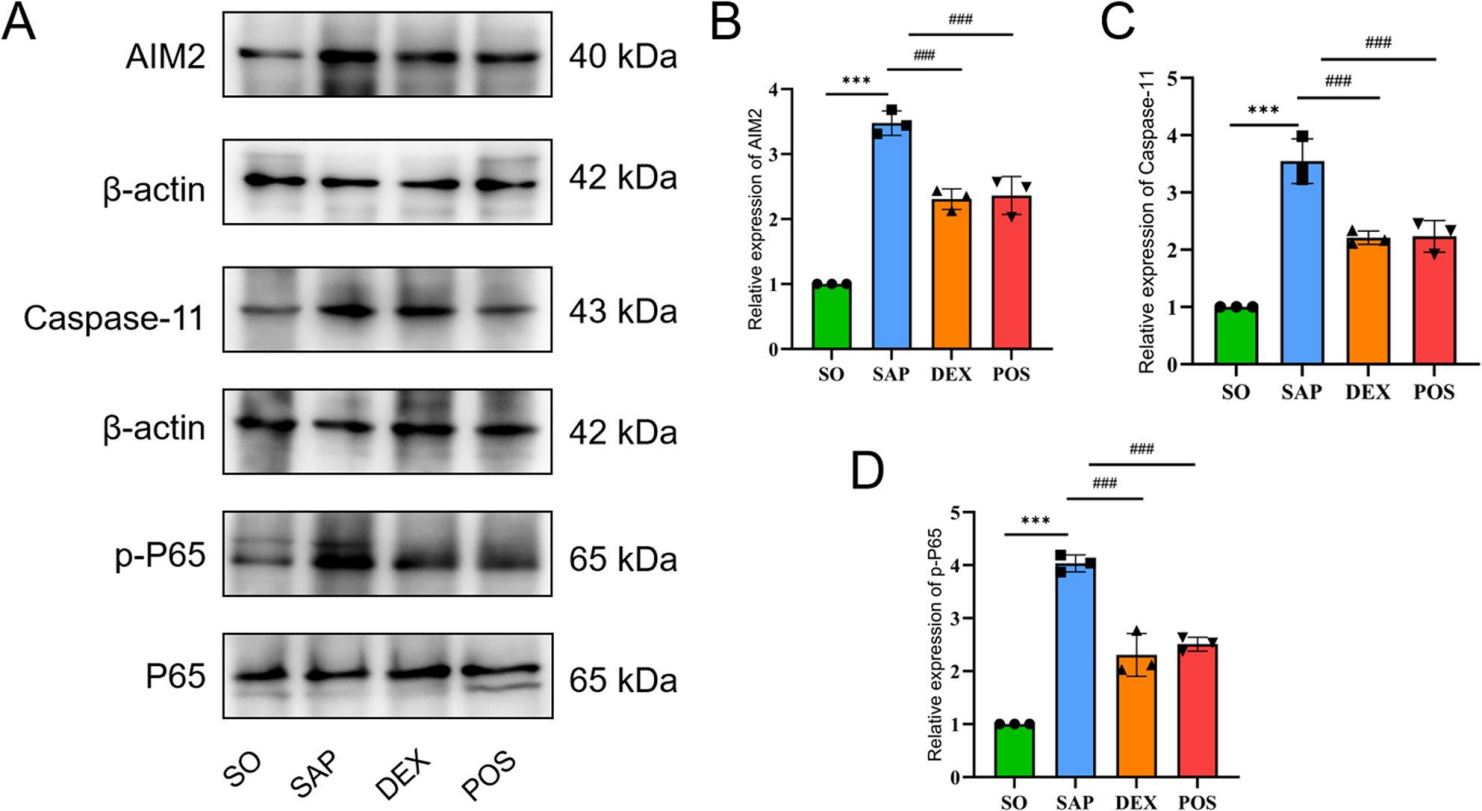
DEX modulates AIM2 inflammasome expression in the lung tissue of SAP rats. **A** Representative Western blot bands for AIM2, Caspase-11, p-P65, and P65 protein expression in lung tissue. **B**-**D** Semi-quantitative analysis of gray values for each protein (*n* = 3). Data are presented as mean ± standard deviation. ^***^ *P*-value < 0.001 vs. SO group, ^###^*P*-value < 0.001 vs. SAP group

**Fig. 7:**
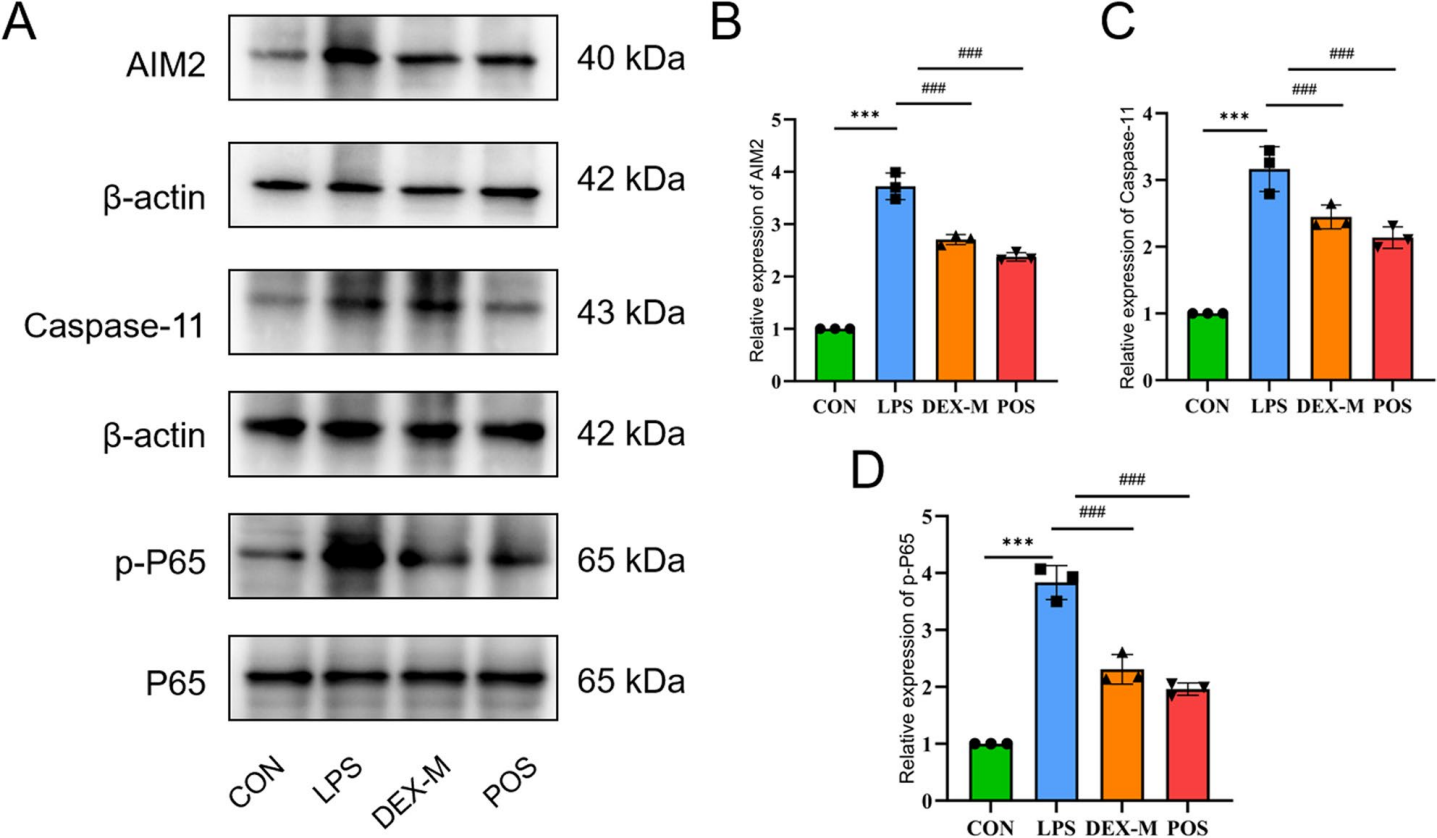
DEX modulates AIM2 inflammasome expression in HUVECs. **A** Representative Western blot bands for AIM2, Caspase-11, p-P65, and P65 protein expression in HUVEC cells. **B**-**D** Semi-quantitative analysis of gray values for each protein (*n* = 3). Data are presented as mean ± standard deviation. ^***^P-value < 0.001 vs. CON group, ^###^*P*-value < 0.001 vs. LPS group

To validate the core targets identified above, key molecules of the AIM2 inflammasome pathway were assessed at both tissue and cellular levels. Western blot analysis revealed that, compared with the SO group, the ratios of AIM2, Caspase-11, and p-P65/P65 were increased in the lung tissue of SAP rats. DEX treatment effectively reversed the upregulation of these molecules (Fig. 6). Due to the complexity of SAP-ALI, no ideal in vitro model currently exists. As LPS accumulates in the circulation and lungs of SAP rats, LPS-stimulated HUVECs have been used to mimic this condition [23]. Thus, we adopted this model in the present study. In vitro, LPS stimulation of HUVECs also activated the AIM2 inflammasome pathway, whereas DEX pre-treatment inhibited the expression of AIM2, Caspase-11, and activation of the p-P65 (Fig. 7). These in vitro and in vivo findings are consistent and collectively demonstrate that DEX confers organ protection by inhibiting AIM2 inflammasome and associated signaling pathways.

### Knocking down AIM2 inhibits LPS-induced HUVEC apoptosis

**Fig. 8:**
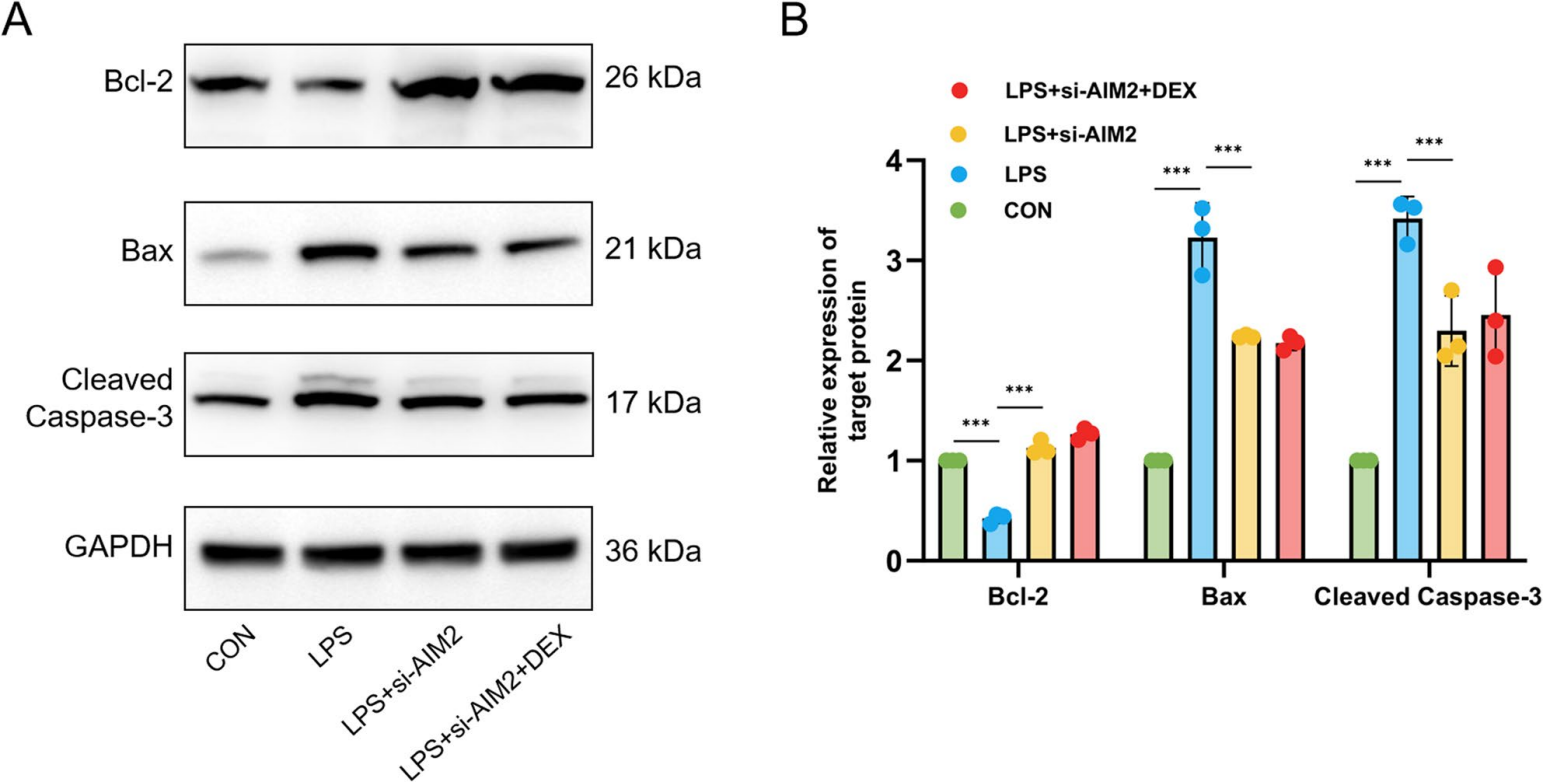
Knocking down AIM2 inhibits LPS-induced HUVEC apoptosis. **A** Representative western blot bands for Bax, Bcl-2, and Cleaved Caspase-3 protein expression in HUVECs. **B** Semi-quantitative analysis of gray values for each protein (*n* = 3). Data are presented as mean ± standard deviation. ^***^ *P*-value < 0.001

Through WB analysis, it was demonstrated that LPS stimulation significantly induced the expression of Bax and cleaved Caspase-3 proteins in HUVECs, while down-regulating the expression of Bcl-2, suggesting apoptosis of HUVECs. However, upon AIM2 knockdown, the expression levels of these proteins were largely restored to baseline, suggesting that AIM2 plays a critical role in LPS-induced apoptotic signaling (Fig. 8). Notably, additional treatment with DEX did not further suppress apoptosis, implying that the protective effect of DEX may be dependent on AIM2 expression.

## Discussion

Because of their complicated pathophysiology and the current dearth of targeted, efficient therapies, SAP and the distal organ damage, particularly ALI, present serious therapeutic problems. This work systematically shows that DEX has a major protective impact against APALI by combining in vitro and in vivo trials with bioinformatics analysis.

DEX’s therapeutic effectiveness was confirmed in rats. In addition to lowering blood amylase activity and the lung tissue wet-to-dry weight ratio, DEX treatment dramatically improved pathological damage in the pancreatic and lung tissues of SAP rats. DEX also significantly reduced blood levels of important pro-inflammatory cytokines, including as TNF-α and IL-1β. These findings demonstrate that DEX has anti-inflammatory effects and imply that its actions could include modification of the inflammasome pathway, given the maturation and release of IL-1β are characteristics of inflammasome activation.

To explore the molecular targets underlying the protective effect of DEX, we used RNA-seq to identify its action targets. We have successfully constructed the “DEX Target Gene Set”, which represents the core gene network that is abnormally activated in SAP and can be effectively rescued by DEX. Enrichment analysis revealed that the “DEX target gene set” was associated with the NOD-like receptor signaling pathway, Toll-like receptor signaling pathway, and Cytosolic DNA-sensing. AIM2 is a pattern recognition receptor capable of sensing double-stranded DNA in the cytoplasm [24, 25]. The inflammasome formed upon its assembly is a key mediator of caspase-1-dependent pyroptosis and IL-1β/IL-18 maturation. Interestingly, molecular docking simulations showed that DEX molecules could stably bind to the active pocket of the AIM2 protein. This suggests that DEX may directly bind to the AIM2 protein.

Analysis of public single-cell RNA sequencing data revealed that the “DEX target gene set” showed significantly altered gene set scores in pulmonary vascular ECs in the ALI model. ECs are essential for maintaining the integrity of the alveolar-capillary barrier, and their pyroptosis directly increases vascular permeability, promotes inflammatory cell infiltration, and leads to tissue edema, serving as a central mechanism in ALI. Immunohistochemical analysis demonstrated that DEX significantly downregulated VCAM-1 and MMP9 expression in lung tissue. VCAM-1 facilitates endothelial cell activation and leukocyte adhesion, while MMP9 contributes to extracellular matrix degradation and tissue injury. The downregulation of these molecules reflects DEX-mediated suppression of endothelial inflammation and restoration of barrier function. Results from western blot analyses showed that DEX significantly inhibited the expression of AIM2, caspase-11, and the p-P65 in lung tissue from SAP rats and in LPS-stimulated HUVECs. It is worth noting that after the AIM2 gene was knocked down, LPS was unable to induce excessive apoptosis in HUVECs, and the stimulation by DEX did not further inhibit apoptosis. This suggests that the protective effect of DEX may depend on the expression of AIM2.

**Fig. 9:**
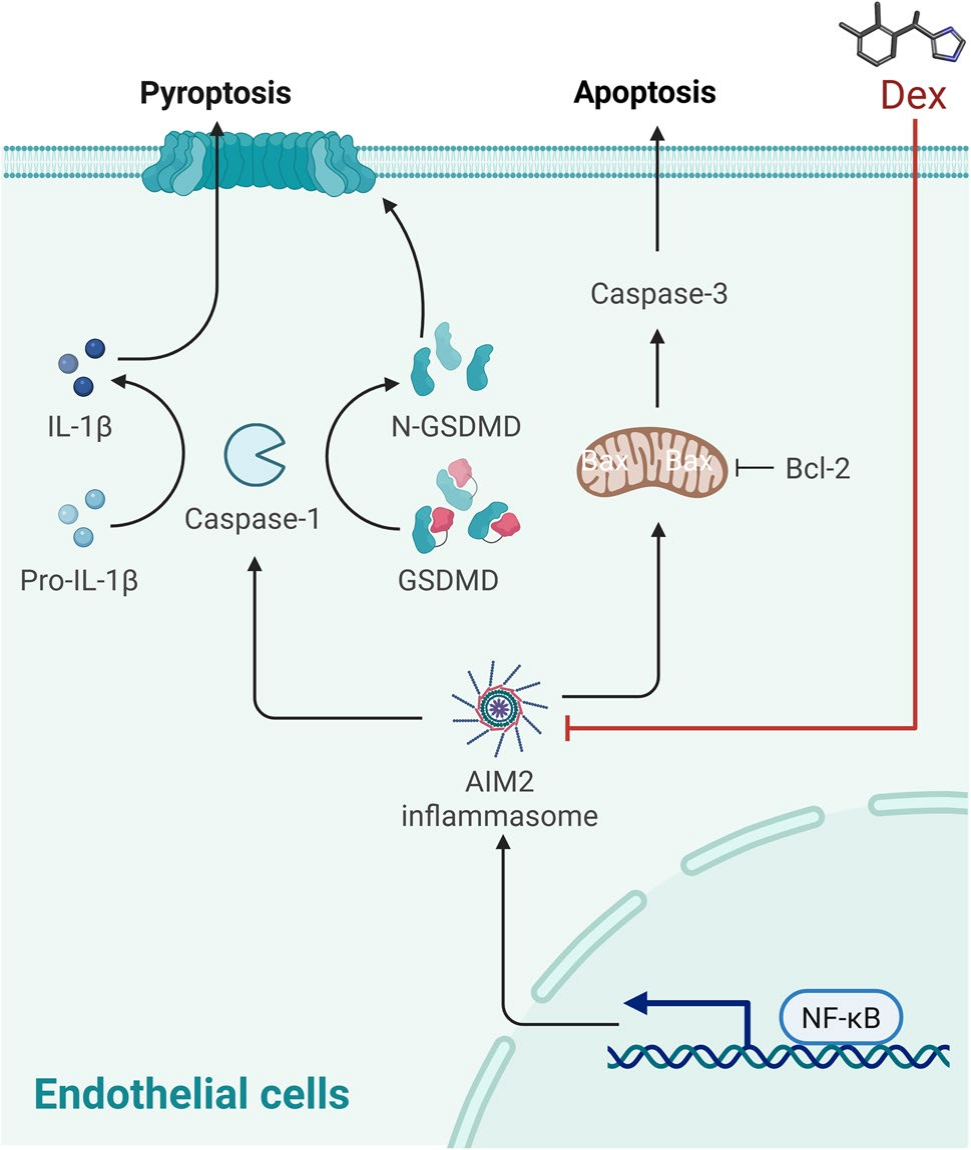
DEX may inhibit endothelial cell pyroptosis and apoptosis by suppressing AIM2 inflammasome activation

Based on all the above findings, we propose that, AP may induce the activation of AIM2 inflammasome in the rat lungs and induce lung injury mediated by endothelial cell pyroptosis and apoptosis. DEX prevents endothelial cell pyroptosis and apoptosis through its anti-inflammatory effect and potential interaction with AIM2, maintains the integrity of the alveolar-capillary barrier, and protects lung tissue (Fig. 9). However, there are still some limitations. Firstly, although molecular docking suggests that DEX can directly bind to AIM2, this must be directly verified using biophysical techniques, such as surface plasmon resonance and point mutation experiments, to confirm the binding site and affinity. Secondly, it is still unclear to what extent the inhibitory effect of DEX, as an α2-AR agonist, on the AIM2 inflammasome depends on this receptor. In the future, the role of α2-AR in this pathway can be clearly defined in cell models by using α2-AR antagonists (or gene knockdown). Thirdly, this study preliminarily confirmed the protective effect of DEX on ECs in vivo and in vitro. However, we established an in vitro model of SAP-ALI using LPS-stimulated HUVECs. While useful, this model does not fully recapitulate the complex in vivo pathology. Future studies could employ organ-on-a-chip platforms for more physiologically relevant investigation. Although we discovered that knocking down AIM2 reversed the protective effect of DEX, we have yet to verify this in animal models. Future research might utilize AIM2 knockout mice or siRNA delivery to show that AIM2 is required for DEX’s protective effects. This would give more robust evidence for clinical translation. Ultimately, our evaluation of pulmonary damage in rats was based on histological scoring. Subsequent investigations should assess the therapeutic impact of DEX on pulmonary damage in rats utilizing imaging techniques.

## Conclusion

In summary, through multi-omics integration and experimental verification, this study has clarified a novel mechanism by which DEX may alleviate pyroptosis of ECs by targeting and inhibiting AIM2 inflammasomes, thereby improving APALI.

## Supplementary Information

Supplementary Material 1: Supplementary Figure S1. Verification of AIM2 knockdown efficiency by qRT-PCR. Supplementary Figure S2. The PPI network identifies core targets of DEX action. Supplementary Figure S3. Enrichment analysis and molecular docking.

## Data Availability

The data and materials used in this study may be provided at the reasonable request of the corresponding author.

## Abbreviations

AIM2: Absent in Melanoma 2
ALI: Acute lung injury
ALT: Alanine aminotransferase
AP: Acute pancreatitis
AST: Aspartate aminotransferase
DEX: Dexmedetomidine
ECs: Endothelial cells
EPC: Edge Percolated Component
GO: Gene ontology
GSEA: Gene set enrichment analysis
H&E: Hematoxylin and eosin
HUVECs: Human umbilical vein endothelial cells
IL-1β: Interleukin-1β
KEGG: Kyoto Encyclopedia of Genes and Genomes
LPS: Lipopolysaccharide
MCC: Maximum Closeness Centrality
MNC: Maximum Neighborhood Component
MMP9: Matrix metalloproteinase-9
POS: Positive control
PPI: Protein-protein interaction
SAP: Severe acute pancreatitis
SO: Sham-operation
STC: Sodium taurocholate
TNF-α: Tumor necrosis factor-α
VCAM-1: Vascular cell adhesion molecule-1
W/D: Wet-to-dry
