# Supplementary material for: Dexmedetomidine may alleviate severe acute pancreatitis-associated lung injury by targeting the AIM2 inflammasome in endothelial cells
Source: BMC Anesthesiol. 2026 Jun 1;26:463. doi: 10.1186/s12871-026-03917-6 (PMC13435787; doi:10.1186/s12871-026-03917-6)
Supplement: Supplementary file 1 [file 12871_2026_3917_MOESM1_ESM.docx]

**Supplementary Figure S1. Verification of AIM2 knockdown efficiency by qRT-PCR.**

**Supplementary Figure S2. The PPI network identifies core targets of DEX action.** (A) Venn diagram illustrating screening of the DEX target gene set. (B-D) Core nodes of the PPI network were identified using the EPC (B), MCC (C), and MNC (D) algorithms.

**Supplementary Figure S3. Enrichment analysis and molecular docking.** (A) Bubble chart of GO functional enrichment analysis for the DEX target gene set. (B) Bubble chart of KEGG pathway enrichment analysis for the top 15 DEX target genes. (C) Molecular docking simulation depicting the binding mode of DEX to the AIM2 protein.
